# 骨桥蛋白及其受体CD44v对肿瘤侵袭调控的研究进展

**DOI:** 10.3779/j.issn.1009-3419.2015.11.11

**Published:** 2015-11-20

**Affiliations:** 300060 天津，天津医科大学肿瘤医院肺部肿瘤科，国家肿瘤临床医学研究中心，天津市“肿瘤防治”重点实验室，天津市肺癌诊治中心 Department of Lung Cancer, Tianjin Medical University Cancer Institute & Hospital, National Clinical Research Center of Cancer, Key Laboratory of Cancer Prevention and Therapy, Tianjin 300060, China

**Keywords:** 骨桥蛋白, CD44v, 信号通路, 肿瘤演进, Osteopontion, CD44v, Signaling pathways, Tumor progression

## Abstract

骨桥蛋白(osteopontion, OPN)是胞外基质中具有多种功能的分泌型糖蛋白，既可作为一种粘附蛋白，参与肿瘤细胞粘附、迁移；又可作为一种细胞因子，促进肿瘤血管形成、逃避免疫监视及抑制细胞凋亡。CD44v糖蛋白是细胞表面粘附分子之一，介导细胞-基质和细胞-细胞间相互作用。近年来大量研究表明OPN及其受体CD44v促进肿瘤侵袭转移，其血清水平与多种肿瘤的预后相关。因此，阐明OPN在肿瘤演进的分子机制及其信号通路有助于寻找一种新颖抗癌疗法。

## Abstract (en)

Osteopontin (OPN) is a multifunctional, extracellular matrix-associated, secretory, glyco-protein. It can be used as an adhesion protein involved in tumor cell adhesion, migration; but also as a cytokine, promoting tumor angiogenesis, evading immune surveillance and inhibiting cellular apoptosis. CD44v glycoprotein, is one of the cell surface adhesion molecule that mediates cell-matrix and cell-cell interactions. Extensive research has suggested the important role of OPN in regulating signaling pathways that contribute to tumor progression and metastasis, and the serum level is associated with the prognosis of various malignancies. Therefore, clarifying OPN in the molecular mechanisms of tumor progression and its signaling pathway contributes to seeking a novel anti-cancer therapy.

骨桥蛋白(osteopontion, OPN)和CD44v是近年来研究较热门的肿瘤相关蛋白，OPN是一种分泌型非胶原性富含唾液酸的糖蛋白，在多种组织细胞广泛表达。OPN可通过改变细胞骨架、降解胞外基质、刺激表皮生长因子、淋巴细胞伪装、激活癌基因Ras等途径促进肿瘤细胞的转移；CD44v可通过降解胞外基质、改变细胞形态、免疫逃避等作用促进肿瘤侵袭。目前认为OPN通过结合胞膜上整合素和CD44受体，激活胞内相关信号通路，增加基质金属蛋白酶(matrix metalloproteinase, MMP)、尿激酶型纤溶酶原激活因子(urokinase type plasminogen activator, uPA)、透明质酸(hyaluronic acid, HA)、血管生成因子(vascular endothelial growth factor, VEGF)等表达，促进肿瘤侵袭转移^[1, 2]^。最近我们对非小细胞肺癌的研究^[3, 4]^表明OPN及其受体CD44v6与肿瘤侵袭转移密切相关。

## 1 OPN的结构

人*OPN*基因定位于染色体4ql3，约8 kb的单拷贝基因，由7个外显子(其中6个包含编码序列)和6个内含子构成并且发现其外显子有3个单核苷酸多肽性区域；*OPN*基因启动子区域包含一个TATA盒、一个反向的CCAAT盒、一个GC盒及多种转录因子的结合位点^[5]^。

OPN是一种带负电荷具有亲水性的分泌型酸性糖蛋白，其相对分子量约44 kDa-75 kDa，具体大小主要由翻译后修饰程度决定；约含300个氨基酸残基，其中天冬氨酸、丝氨酸和谷氨酸残基约占总氨基酸的一半^[6]^。人OPN通过转录水平的选择性剪接产生三种亚型：OPN-a、OPN-b、OPN-c，其中全长形式的OPN-a，包含所有外显子；OPN-b mRNA序列缺少外显子5；而OPN-c mRNA序列缺少外显子4。三种亚型在多种类型的肿瘤中均有表达，但尚未阐明具体病理意义及功能^[7]^。OPN一般由信号肽序列、7个-10个连续天冬氨酸序列、精氨酸-甘氨酸-天冬氨酸(Arg-Glu-Asp, RGD)结构域、整合素α9β1/α4β1结构域、凝血酶切割位点、钙离子结合位点和肝素结合位点构成。OPN可被凝血酶裂解为2个功能区：N端片段经磷酸化后依赖RGD基序与整合素受体结合，介导细胞的迁移、趋化和粘附，同时具有抗凋亡和免疫逃逸功能；C端片段不依赖RGD基序与CD44受体结合，介导细胞内信号传导，提高粘附功能，并参与免疫逃逸和淋巴细胞归巢^[1]^。OPN经凝血酶切割后，同时暴露非依赖RGD基序与α9β1/α4β1结合的SVVYGLR粘附序列^[8]^。

## 2 OPN的主要受体

### 2.1 整合素家族

整合素是由α和β亚基通过非共价键形式连接而成一种细胞表面糖蛋白，其中与肿瘤转移相关的主要是α9β1和αvβ3。OPN主要通过识别RGD结构域与整合素受体结合，介导细胞粘附与迁移，并改变宿主免疫力^[9]^；而α9β1对RGD无依赖性，可能通过识别SVVYGLR位点介导细胞与细胞和细胞与基质间的相互作用，在血管生成中发挥作用^[8]^。

### 2.2 CD44结构与功能

CD44是一种高度异质性细胞表面跨膜糖蛋白，广泛分布于炎细胞、上皮细胞、软骨细胞、纤维母细胞及肿瘤细胞等；其相对分子量约80 kDa-200 kDa，主要由选择性剪切和蛋白修饰程度决定。CD44胞膜外区具有与透明质酸结合的能力，也是其发挥生物学功能的重要区域；跨膜区由21个疏水性氨基酸组成；胞质区可被蛋白激酶C磷酸化，参与细胞内信号传导。人CD44基因位于11号染色体短臂，由20个外显子构成。根据外显子的表达方式不同可分为标准型CD44(CD44s)和变异型CD44(CD44v)。某些CD44v，如CD44v6已作为肝癌、乳腺癌、结直肠癌及胃部肿瘤转移判断的标志。与整合素家族不同，CD44主要通过氨基酸缩聚反应(CD44v6)或肝素桥接(CD44v3, CD44v9)与OPN结合产生趋化、抗凋亡及粘附等作用^[10]^。

CD44v主要出现在机体的病理过程，其中CD44v6在肿瘤进展中发挥重要作用，其机制可能是：①促进肿瘤细胞与基底膜粘附蛋白及Ⅳ型胶原酶的粘附，为胞外基质的降解创造条件；②作为受体介导HA与骨架蛋白相结合，参与细胞伪足的形成，引起细胞形态和游走性改变，促使癌细胞转移；③CD44v6高表达的癌细胞可获得淋巴细胞伪装，逃避免疫系统的识别和杀伤；④促进肿瘤间质细胞释放蛋白水解酶^[11]^。

## 3 OPN在肿瘤侵袭、转移中的作用

肿瘤细胞通过自分泌产生OPN，促进自身粘附、侵袭、转移，与肿瘤细胞恶性表型密切相关。包括：对细胞骨架进行调节，降解胞外基质，参与肿瘤细胞粘附、趋化和迁移；促进肿瘤血管生成；逃避宿主免疫反应；抑制肿瘤细胞凋亡等。

### 3.1 调节细胞骨架，降解胞外基质，促进细胞运动

OPN通过RGD序列与整合素受体结合，引起聚集粘附激酶磷酸化，进而激活Ras和丝裂原激活蛋白激酶(mitogen-activated protein kinase, MAPK)信号转导途径，促进细胞内OPN与HA-CD44-ERM(埃兹蛋白/根蛋白/膜突蛋白)结合成复合体，改变细胞骨架，参与成纤维细胞的迁移、巨噬细胞的激活和肿瘤细胞的转移^[12]^。有研究^[13]^表明Ezrin蛋白可直接或间接与CD44v6等结合，进一步来调节肿瘤细胞的粘附性，影响肿瘤的侵袭、转移。OPN与整合素受体结合，通过核因子κB、AKT等信号转导途径促进MMP2、MMP9、uPA的表达，从而降解胞外基质^[10]^；还可通过c-Src途径活化细胞粘附分子，促进肿瘤细胞趋化粘附和迁移^[14]^。

### 3.2 促进肿瘤血管生成，逃逸宿主免疫，抑制细胞凋亡

OPN可使VEGF表达上调，而VEGF可通过激活PI3K/AKT等途径刺激OPN的表达。Ito等^[8]^研究证明OPN的SVVYGLR序列能有效促进血管内皮细胞的粘附和迁移。Chakraborty等^[15]^研究表明OPN结合整合素受体，启动肿瘤血管的生成，在此过程中依赖于VEGF的作用。Dai等^[16]^发现OPN通过激活内皮细胞PI3K/AKT和ERK1/2信号诱导血管生成。最近报道OPN可降低环氧酶2活性、抑制巨噬细胞NO合酶的分泌，避免NO对肿瘤细胞的毒性作用，从而使癌细胞在血液循环中得以存活^[17]^。OPN RGD区域通过激发NF-κB通路和促进聚集粘附激酶磷酸化来抑制细胞凋亡^[9]^。

### 3.3 促进上皮间质转化(epithelial-mesenchymal transition, EMT)

越来越多证据^[18]^表明肿瘤细胞发生EMT后能获得侵袭表型，癌细胞进入周边基质，形成有利于转移形成的微环境，并最终促使转移的发生。有研究^[19]^表明OPN上调EMT进而促进乳腺癌细胞侵袭转移。Bhattacharya等^[20]^研究肝癌移植小鼠模型时发现OPN通过调控EMT促进肿瘤生长。

## 4 OPN-CD44v相互作用与肿瘤侵袭转移

Gao等^[21]^采用蛋白印迹法检测HepG2肝癌细胞株中OPN对CD44v6的影响，结果发现OPN上调CD44v6在胞膜上的表达，OPN这种作用的实现依赖于酪氨酸激酶活性和与αvβ3结合的能力；同时随着CD44v6的升高，癌细胞粘附基质的能力增强从而增加肝癌的转移潜能。另有研究^[22]^表明CD44+结直肠癌细胞能促进巨噬细胞OPN的分泌，进而通过激活JNK通路促进癌细胞生长；敲除CD44后，癌细胞分泌的OPN减少。Kumar等^[23]^研究表明过表达的OPN能促进宫颈癌细胞生长；短发夹RNA沉默OPN表达，OPN-CD44调控的MAPK激酶依赖性P38未激活进而抑制癌细胞生长。

## 5 OPN调控肿瘤的相关信号途径

### 5.1 PI-3K/Akt/NF-κB通路(磷脂酞肌醇3激酶/蛋白激酶B/核因子通路)

AKT作为PI3K下游靶蛋白，是信号转导途径中的重要激酶。PI3K-AKT介导细胞侵袭和血管生成作用^[24]^。OPN与整合素αvβ3结合后可诱导NF-κB活化从而促进MMP2和uPA的表达，诱发肿瘤血管的生成及胞外基质的降解^[5]^；OPN通过结合α9β1增强NF-κB依赖的环氧酶2的表达促进肿瘤血管形成^[17]^。

### 5.2 c-Src/EGFR/ERK通路(c-Src/表皮生长因子受体/胞外信号调节激酶通路)

c-Src属于非受体型蛋白酪氨酸激酶家族，参与调控整合素受体的下游信号通路。OPN与αvβ3结合可诱导c-Src的活化和EGFR的磷酸化，进而促进胞外信号调节激酶的活化，将信号传导至胞核；c-Src还可诱导核转录因子激活蛋白1的活化及uPA的分泌，促进胞外基质的降解、肿瘤血管的生成。此外，c-Src还能活化PI3K、Ras-MAPK、磷脂酶C等激酶和细胞粘附分子，调控细胞的粘附及转移^[24-26]^。

### 5.3 NIK/MAPK/IKK途径(核因子诱导激酶/丝裂原激活蛋白激酶/ NF-κB抑制蛋白激酶途径)

NIK属于丝裂原激活蛋白激酶家族，在基因表达调控和胞质功能活动中发挥重要作用。OPN与αvβ3结合后，NIK发生磷酸化，活化的NIK与IKKα或IKKβ相互作用，激活IKK进而促发NF-κB通路。此外，活化的NIK还参与OPN诱导ERK激酶MEK1的磷酸化，活化的MEK1又可诱导转录因子AP1的活化，最终促进uPA、MMP的分泌^[27]^。

### 5.4 OPN-CD44转导途径

OPN通过与CD44受体相互作用激活PLC-γ/PKC/PI3k信号通路，促进细胞存活和细胞运动。OPN与CD44结合，活化第二信使PLC-γ，促使蛋白激酶C磷酸化而激活PI3K，诱导其下游分子Akt的磷酸化，进而调控细胞周期、促进细胞迁移；此外，OPN加强活化的Akt与IKK相互作用，诱导IKK活化，降解NF-κB抑制剂IκBα，从而促发NF-κB通路。Teramoto等^[27]^发现OPN与CD44结合并同时高表达于肿瘤组织，通过OPN-CD44-Ras-MAPK途径促进癌细胞趋化粘附和迁移，激活的Ras活化Raf(MAPK kinase kinase, MAPKKK)进而激活MEK(MAPK kinase, MAPKK)最终导致胞外信号调节激酶ERK激活，从而在肿瘤复发与转移中起重要作用。研究^[28]^表明OPN通过结合其受体CD44激活c-jun-NH2激酶信号，促进大肠癌细胞的克隆形成。

## 6 展望

尽管OPN参与肿瘤侵袭转移的具体分子机制仍不明确，OPN作为临床筛查肿瘤、监测复发、判断预后的指标己成共识；进一步阐明OPN及其受体与肿瘤细胞发生、浸润及转移的关系，对开创肿瘤临床诊治的新局面非常必要。
